# Supplementary figures and images for: Cortical activity associated with the maintenance of balance during unstable stances
Source: PeerJ. 2024 Apr 30;12:e17313. doi: 10.7717/peerj.17313 (PMC11067896; doi:10.7717/peerj.17313)

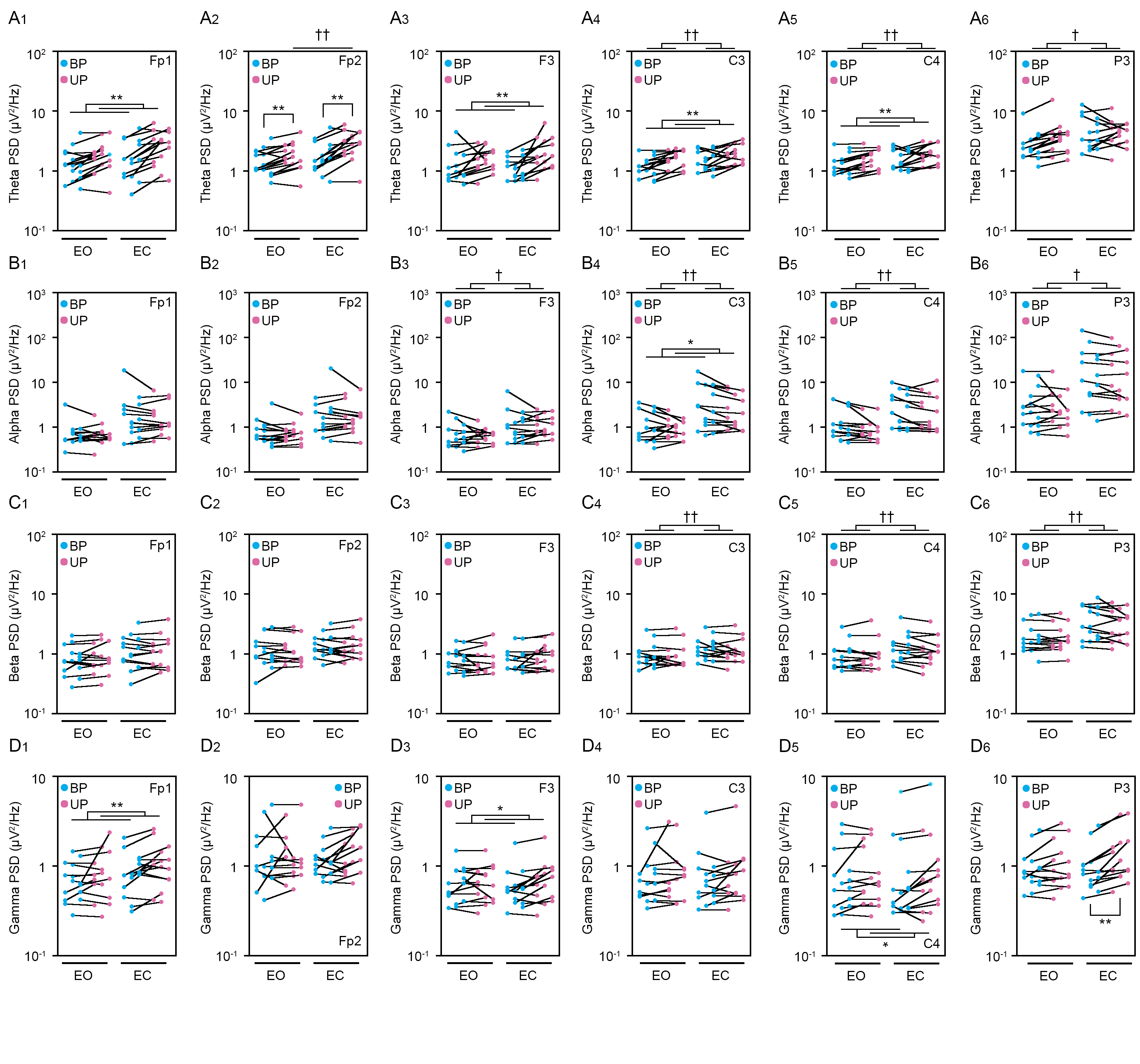

Supplement: Supplemental Information 1 — (A) Alterations in theta power spectral densities (PSDs) in Fp1 (A1), Fp2 (A2), F3 (A3), C3 (A4), C4 (A5), and P3 (A6) channels during BP (cyan) and UP (magenta) standing periods under EO and EC conditions. (B) Alterations in alpha PSDs in Fp1 (B1), Fp2 (B2), F3 (B3), C3 (B4), C4 (B5), and P3 (B6) channels during BP (cyan) and UP (magenta) standing periods under EO and EC conditions. (C) Alterations in beta PSD in Fp1 (C1), Fp2 (C2), F3 (C3), C3 (C4), C4 (C5), and P3 (C6) channels during BP (cyan) and UP (magenta) standing periods under EO and EC conditions. (D) Alterations in gamma PSDs in Fp1 (D1) and Fp2 (D2), F3 (D3), C3 (D 4 ), C4 (D5), and P3 (D6) channels during BP (cyan) and UP (magenta) standing periods under EO and EC conditions. Plots represent the data obtained from individual participants. Number of participants: n = 15. Statistical differences were analyzed using repeated two-way ANOVA test. Abbreviations: AP, anteroposterior; BP, bipedal; EC, eye closed; EEG, electroencephalogram; EO, eye open; PSD, power spectral density; UP, unipedal. Statistical significance: *P < 0.05 (vs. BP), **P < 0.01 (vs. BP), † P < 0.05 (vs. EO), ††P < 0.01 (vs. EO). [file peerj-12-17313-s001.png]

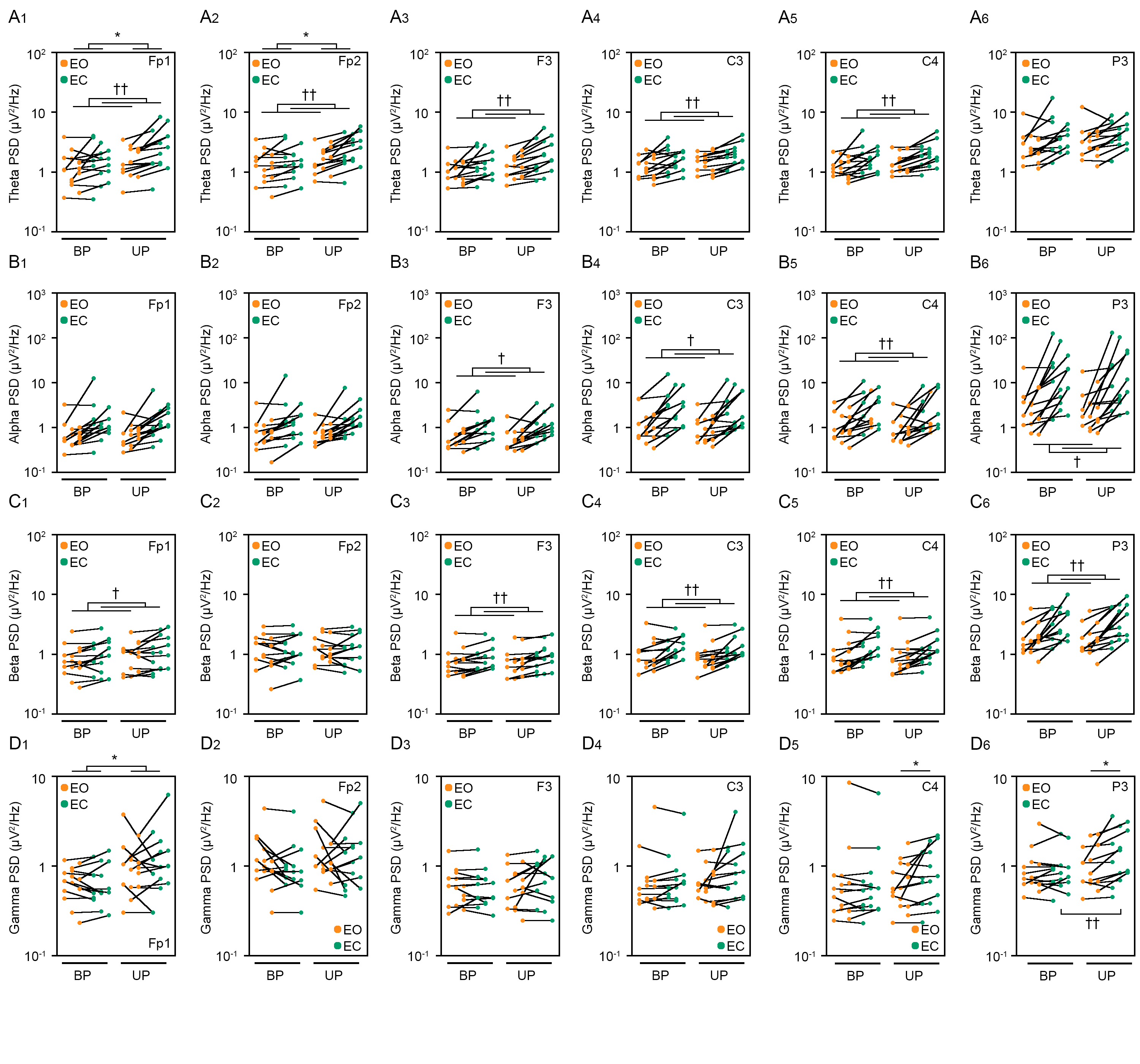

Supplement: Supplemental Information 2 — (A) Alterations in theta power spectral densities (PSDs) in Fp1 (A1), Fp2 (A2), F3 (A3), C3 (A4), C4 (A5), and P3 (A6) channels during BP and UP standing periods under EO (orange) and EC (green) conditions. (B) Alterations in alpha PSDs in Fp1 (B1), Fp2 (B2), F3 (B3), C3 (B4), C4 (B5), and P3 (B6) channels during BP and UP standing periods under EO (orange) and EC (green) conditions. (C) Alterations in beta PSDs in Fp1 (C1), Fp2 (C2), F3 (C3), C3 (C4), C4 (C5), and P3 (C6) channels during BP and UP standing periods under EO (orange) and EC (green) conditions. (D) Alterations in gamma PSDs in Fp1 (D1), Fp2 (D2), F3 (D3), C3 (D4), C4 (D5), and P3 (D6) channels during BP and UP standing periods under EO (orange) and EC (green) conditions. Plots represent the data obtained from individual participants. Number of participants: n = 15. Statistical differences were analyzed using repeated two-way ANOVA test. Abbreviations: AP, anteroposterior; BP, bipedal; EC, eye closed; EEG, electroencephalogram; EO, eye open; PSD, power spectral density; UP, unipedal. Statistical significance: *P < 0.05 (vs. BP), † P < 0.05 (vs. EO), ††P < 0.01 (vs. EO). [file peerj-12-17313-s002.png]
